# Hierarchical fusion detection algorithm for sleep spindle detection

**DOI:** 10.3389/fnins.2023.1105696

**Published:** 2023-03-09

**Authors:** Chao Chen, Jiayuan Meng, Abdelkader Nasreddine Belkacem, Lin Lu, Fengyue Liu, Weibo Yi, Penghai Li, Jun Liang, Zhaoyang Huang, Dong Ming

**Affiliations:** ^1^Academy of Medical Engineering and Translational Medicine, Tianjin University, Tianjin, China; ^2^Key Laboratory of Complex System Control Theory and Application, Tianjin University of Technology, Tianjin, China; ^3^Department of Computer and Network Engineering, College of Information Technology, United Arab Emirates University, Al Ain, United Arab Emirates; ^4^Zhonghuan Information College Tianjin University of Technology, Tianjin, China; ^5^Beijing Machine and Equipment Institute, Beijing, China; ^6^Department of Rehabilitation, Tianjin Medical University General Hospital, Tianjin, China; ^7^Department of Neurology, Xuanwu Hospital, Capital Medical University, Beijing, China; ^8^Beijing Key Laboratory of Neuromodulation, Beijing, China

**Keywords:** sleep spindle detection, hierarchical fusion detection algorithm, EEG, Morlet wavelet, SVM

## Abstract

**Background:**

Sleep spindles are a vital sign implying that human beings have entered the second stage of sleep. In addition, they can effectively reflect a person’s learning and memory ability, and clinical research has shown that their quantity and density are crucial markers of brain function. The “gold standard” of spindle detection is based on expert experience; however, the detection cost is high, and the detection time is long. Additionally, the accuracy of detection is influenced by subjectivity.

**Methods:**

To improve detection accuracy and speed, reduce the cost, and improve efficiency, this paper proposes a layered spindle detection algorithm. The first layer used the Morlet wavelet and RMS method to detect spindles, and the second layer employed an improved k-means algorithm to improve spindle detection efficiency. The fusion algorithm was compared with other spindle detection algorithms to prove its effectiveness.

**Results:**

The hierarchical fusion spindle detection algorithm showed good performance stability, and the fluctuation range of detection accuracy was minimal. The average value of precision was 91.6%, at least five percentage points higher than other methods. The average value of recall could reach 89.1%, and the average value of specificity was close to 95%. The mean values of accuracy and F1-score in the subject sample data were 90.4 and 90.3%, respectively. Compared with other methods, the method proposed in this paper achieved significant improvement in terms of precision, recall, specificity, accuracy, and F1-score.

**Conclusion:**

A spindle detection method with high steady-state accuracy and fast detection speed is proposed, which combines the Morlet wavelet with window RMS and an improved k-means algorithm. This method provides a powerful tool for the automatic detection of spindles and improves the efficiency of spindle detection. Through simulation experiments, the sampled data were analyzed and verified to prove the feasibility and effectiveness of this method.

## 1. Introduction

Sleep spindles refer to the recognizable 11–16 Hz sinusoidal periodic pulse sequence on an electroencephalogram (EEG) during sleep. According to the American Academy of Sleep Medicine (AASM) (Iber et al., 2007), the spindles mainly occur in stage two of Non-Rapid-Eye-Movement (NREM) sleep (Schilling et al., 2018; Chriskos et al., 2019). Spindle detection plays a crucial role in sleep staging research and clinical disease diagnosis (Dehnavi et al., 2019; Zhang et al., 2020). Studies have found that the number and density of spindles are associated with many diseases (Fogel and Smith, 2011), such as Parkinson’s disease (Latreille et al., 2015), Alzheimer’s disease, major depression, autism, insomnia, and schizophrenia (Limoges et al., 2005; Keshavan et al., 2011; Astori and Luthi, 2013; Davies et al., 2016; Spironelli et al., 2020). In addition, the function of sleep spindles is associated with human intelligence and sleep-dependent memory consolidation (Fogel et al., 2007; Ujma et al., 2014). Notably, spindles are a reflecting functional brain-state biomarker and have solid supplementary diagnostic value (Zhao et al., 2017; Mensen et al., 2018).

Currently, in clinical diagnosis, spindle detection mainly depends on the subjective experience of doctors, the so-called gold standard of spindle detection (Dakun et al., 2015). Generally, the manual detection of spindles allows many experts to select the spindles simultaneously. It is challenging to detect sleep spindles with this method due to the high detection cost and longer detection time (Lacourse et al., 2019). Thus, Wamsley et al. (2012) explored a method to detect spindles using the wavelet transform automatically and found an overall number and density of 62.5%, a result that was far from ideal. Athanasios and Clifford (2015) and others proposed a probabilistic wavelet estimation algorithm based on the wavelet algorithm for the automatic detection of spindles. However, due to the spindles’ irregularities, the performance in recall rate was not ideal, with a minimum of 14.4% and a maximum of 83.2%. Hence, the stability of the recall rate was poor. Martin (Martin et al., 2012) et al. used the window root mean square (RMS) method to detect the spindle density of young and older people by calculating the RMS value. The accuracy was only 72%, but the recall rate was 83%. Furthermore, concerning age, it was found that the density, duration, and amplitude of spindles in young subjects were greater than those in older subjects, and age factors affected the detection of spindles. Mporas et al. (2013) proposed the hidden Markov model (HMM) and support vector machine (SVM) to process EEG signals. Through the fusion of HMM and SVM (HMM&SVM), the output recognition spindle results were combined to extract the final sleep spindle detection results. The average performance regarding precision was 88%, and recall was 76%, but the process was complex, and the operation was cumbersome, bringing great inconvenience to the experiment. Lacourse et al. (2019) proposed a detection method for spindles, also known as A7. The recall rate of this method reached 68%, with no crucial change compared with the average value of 72.7% within and between experts in sleep spindle detection (Devuyst et al., 2006).

The performance index of the above algorithms is in the range of 60–70%, and thus their performance is not ideal. Currently, there is a shortage of public databases for spindle detection, and the lack of databases has led to difficulties in validating the stability of different detection algorithms (Wendt et al., 2015). The performance of spindle detection can be further improved on the original basis.

Herein, a new notion of hierarchical fusion algorithm is proposed to improve the defects of expert manual detection and the automatic detection of spindles. It addresses the advantages and disadvantages of the automatic detection methods of sleep spindles by combining the advantages of the spindle detection method and overcoming the shortcomings of previous methods to detect spindles. The Morlet wavelet and RMS algorithms are used as the first layer of the basic algorithm. After fusing the results of the two automatic detection methods, the k-means algorithm in the second layer is used for clustering to get the final result. The Morlet wavelet detection method, window RMS detection method, HMM&SVM algorithm, and the newly proposed hierarchical fusion algorithm are compared in the detection results. The data results optimized by the hierarchical fusion algorithm substantially improve the performance of spindle automatic detection. In order to improve the efficiency and accuracy of spindle detection, we propose a new spindle detection algorithm, which combines the Morlet wavelet detection algorithm, RMS algorithms, and k-means algorithm. The average accuracy of this method is 91.6%, at least five percentage points higher than other methods.

## 2. Materials and methods

### 2.1. Data sources and methods

The experimental data came from the sleep monitoring room of Beijing Xuanwu Hospital. This experiment was designed to collect sleep data from 20 subjects with sleep disorders, all of whom were between 20 and 40 years old in order to avoid the effect of age differences on the number of spindles. Their average age was 31 years old, 11 participants were female, and they were all recruited from the community. Twenty subjects were scored on the Pittsburgh Sleep Quality Index before EEG acquisition. Those with a score greater than or equal to 11 were patients with sleep disorders. Table 1 shows the Pittsburgh Sleep Quality Index scores of the 20 subjects in this paper. The higher the score, the worse the sleep quality.

**TABLE 1:** Pittsburgh sleep quality index score.

Subject	Age	Sex	Score	Subject	Age	Sex	Score
DS1	21	Male	14	DS11	24	Male	12
DS2	35	Male	17	DS12	40	Female	18
DS3	40	Female	19	DS13	31	Female	20
DS4	25	Male	16	DS14	22	Male	16
DS5	34	Female	16	DS15	30	Female	15
DS6	20	Female	18	DS16	37	Female	20
DS7	36	Female	11	DS17	25	Male	18
DS8	29	Male	13	DS18	33	Female	17
DS9	38	Female	16	DS19	34	Male	14
DS10	32	Male	14	DS20	29	Female	16

DS represents subjects with sleep disorders. Subjects with a score greater than or equal to 11 are subjects with sleep disorders.

**FIGURE 1:**
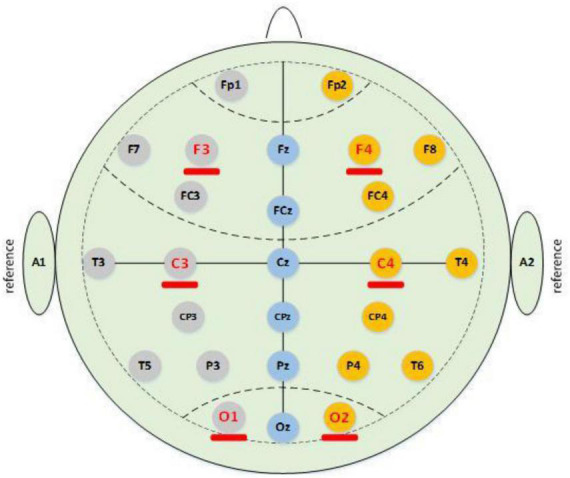
International 10 / 20 standard electrode placement system.

The data collection equipment adopted polysomnography (PSG). The equipment could record many channels simultaneously during the subjects’ sleep, such as EEG, ECG, EOG, EMG, airflow, and oxygen saturation. As shown in Figure 1, the international 10 / 20 standard electrode placement system shows the electrode positions of the relevant EEG signals collected in this paper. Based on the relevant research on the brain regions with frequent spindles, this study collected and analyzed the EEG signals collected from only six channels. Thus, the EEG data of the left and right channels collected in this experiment included F3 \F4 \C3 \C4 \O1 \O2. Here, F3 and F4 are frontal brain regions, C3 and C4 are central brain regions, and O1 and O2 represent the occipital regions. Spindles appear most frequently at these positions, and C3/C4 have the most spindle appearances. The sampling frequency of the EEG signal acquisition equipment used in this experiment was 1,024 Hz.

The data collection for each subject lasted from 9:30 p.m. to 6:30 a.m. The start and end times of the subjects entering sleep were not wholly consistent. Thus, sleep EEG data of up to 8 h from the first entering sleep stage were intercepted based on the stage of sleep for research to facilitate and accurately analyze the characteristic differences in individual and overall sleep spindles in the later stages. Professional sleep researchers operated and guided the monitor used in the sleep monitoring room. To ensure the accuracy of the physiological signal collection, the subjects were required to prohibit the intake of alcohol and the consumption of caffeine, sedatives, hypnotics, and other relevant drugs that could affect data collection 1 week before the sleep monitoring. Before data acquisition, the subjects were required to have taken a bath and cleaned their heads to ensure good contact between the electrodes and the skin to assist the collecting of data as much as possible. Furthermore, the subjects were required to urinate in advance or place a disposable night pot next to the hospital bed to avoid the disproportionate impact of large-scale activities at night on data acquisition. Next, the experimenter recorded the subject’s name, weight, gender, age, and other essential information. The relevant electrode connection points were wiped with a cleaning paste specifically used to clean the electrodes before the electrodes were connected. When the electrode was placed, the accuracy and firmness of the electrode’s position could be secured, and the electrodes were placed in a specified order. After the work preparation, before collection was completed, the monitor was opened to record the data collected by the software. During the acquisition process, the cell phones of the subject and experimenter were turned off to keep the environment quiet to avoid the interference of external environmental sound on the experimental data. After data collection in the morning, the experimenter turned off the equipment and woke up the subject. At this point, the experimental data collection was finished.

The sampling frequency of the experimental data was 1,024 Hz, which was downsampled to 512 Hz during the pre-processing for this paper. In this experiment, the bilateral mastoid was used as the mean reference for re-referencing. The Morlet wavelet-based and RMS automatic detection methods used in this experiment both require band-pass filtering of the data prior to spindle wave detection. The raw EEG signal was pre-processed with band-pass filtering from 5 to 35 Hz prior to automatic spindle wave detection using Morlet wavelets. Pre-processing of the raw data with 11–16 Hz bandpass filtering was conducted before using the RMS algorithm, where the frequency band was chosen based on the standard definition of the spindle frequency distribution.

### 2.2. Proposed algorithm

**FIGURE 2:**
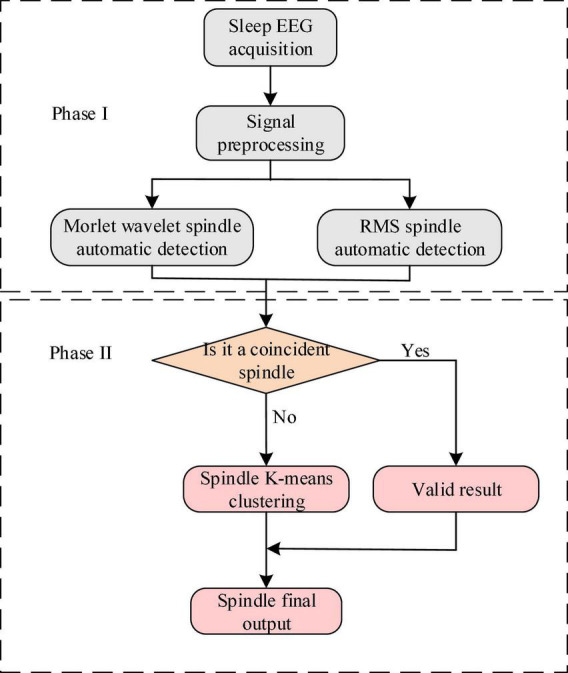
Flowchart of the fusion algorithm.

Figure 2 is the flowchart of the newly proposed hierarchical fusion spindle automatic detection algorithm. Two single detection algorithms, the Morlet wavelet and window RMS were used in the fusion algorithm, merged with the improved k-means algorithm.

The specific steps of the hierarchical fusion algorithm were as follows:

Firstly, the collected sleep EEG signal was pre-processed. The resultant sleep signal was transmitted to the Morlet wavelet and window RMS spindle automatic detector to judge the true and false spindle of the two detectors’ output results.

The Morlet wavelet function was closer to the spindles and more conducive to spindle detection. Spindle detection function based on the Morlet wavelet was defined as:


(1) $$f\,\left(x\right)={\left(\pi \,F_{B}\right)}^{-0.5}\,\exp\left(2\,\pi \,{\mathrm{iF}}_{C}\,x\right)\,\exp\left(-x^{2}/F_{B}\right)$$

Where F_*B*_ is the bandwidth of wavelet transform, F_*B*_ = 2s^2^, and s = n/2πF_*C*_. The value of F_*B*_ depends on the magnitude of the values of *n* and F_*C*_. The *n* represents the number of cycles of the Morlet wavelet. F_*C*_ is the center frequency. Here, set *n* = 7 is a typical default value when balancing the time-frequency domain.

The Morlet wavelet function performed time-frequency conversion on the pre-processed EEG signal, and a threshold function was used to detect the spindle. After all pre-processing, the threshold was defined as 4.5 times the average signal amplitude. The average moving value was calculated using a 0.1 s sliding window to extract the spindle in the frequency band. When the wavelet signal exceeded the threshold and the duration was in the range of 0.5–3 s, it was deemed a spindle. If the distance between the two spindles was less than 1 s and the duration was less than 3 s, the spindle was combined. This detection result was reserved for the fusion of later experiments.

The window RMS algorithm used a linear phase finite impulse response filter for 11–16 HZ band-pass filtering of the EEG original signal in the NREM period of the C3 channel, doubling the order of the filter. The filtered EEG signal was determined using a time window of 0.25 s, and the threshold value was 0.95 times the mean value. The spindle was identified as two consecutive root mean squares calculated time points, exceeding the threshold and lasting between 0.5 and 3 s. The RMS value was calculated every 5 s by employing the following formula:


(2) $$R\,M\,S-A=\sqrt{\frac{\sum_{i=1}^{N}X_{i}^{2}}{N}}$$

RMS-A refers to the root mean square of the spindle wave frequency band. Where X_*i*_^2^ is the square of the amplitude of the sampling point i, and *N* is the number of sampling points within 5 s.

**FIGURE 3:**
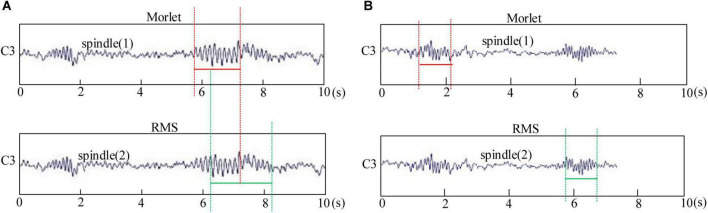
Spindle automatic detection results of the Morlet wavelet and root mean square (RMS). **(A)** Morlet wavelet and RMS detect the coincident spindle. **(B)** Morlet wavelet and RMS detect non-coincident spindle.

Let us repeat the spindle detection link. The spindle detected by the Morlet wavelet and RMS method was divided into coincident and non-coincident spindles. When the spindle detected using the two methods overlapped in the time series, they were considered coincident spindles. As can be seen in Figure 3A, the coincident spindle was regarded as the effective result of the fusion algorithm detection. When the spindle time did not repeat, it was considered a non-coincident spindle, as can be seen in Figure 3B.

This study identified the coincident spindle as the same spindle and directly classified it into the final automatic detection result set. The non-coincident spindle set was treated as the sample of clustering input. After cluster analysis, many non-spindle clusters were removed, and the samples of the remaining clusters and the coincident spindle set were taken as the final output result.

The non-coincident spindle needed to be further analysed and processed by the K-means clustering method. The K-means algorithm was relatively stable, and had a very smooth clustering effect. The K-means clustering algorithm needed to calculate the distance between the points of each cluster. The distance metric commonly used in the k-means algorithm is Euclidean distance.

Euclidean distance (L2):


(3) $$d_{12}=\sqrt{{\left(x_{1}-x_{2}\right)}^{2}+{\left(y_{1}-y_{2}\right)}^{2}}$$

The density was the distance between points to judge the abnormal points. After removing the outliers, the data were put into K-means clustering, improving the accuracy of clustering, reducing the amount of clustering data, and enhancing data processing speed.

The sum of squared errors (SSE) was used as the objective function to evaluate the clustering effect so that the clustering result can obtain the minimum SSE value (Klampanos et al., 2009). The local outlier factor detection method optimized K-means clustering, addressing the detection problem of amplitude affecting the spindle.

The amplitude of the spindles was in the range of 10–60 _μ*V*_. When clustering the non-coincident spindles, the amplitude was used as the input. Five categories were clustered based on the amplitude, and the maximum and minimum were removed. The remaining three categories were used as data for further analysis. After clustering, clusters with many non-spindles were discarded, leaving the actual spindle clusters.

Finally, the final fusion result was the clustering and the coincident spindle.

In this paper, when using the k-means algorithm for clustering, the method of calculating the difference statistics was adopted to select the k value.

### 2.3. Evaluation method

Twenty subjects were tested manually using the gold standard of spindle detection to prove the reliability of the hierarchical fusion spindle automatic detection method. The gold standard adopted was three experts conducting the sample’s artificial spindle detection. The intersection of the three experts’ detection results were determined as spindles here, and the detection results not within the intersection were regarded as non-spindle sets.

Then according to the confusion matrix and the gold standard of spindle detection, the evaluation indexes, such as TPR (recall), specificity, accuracy, precision, and F1-score, were calculated to evaluate the detector’s performance.

The following was the meaning and relevant calculation formula of these evaluation indicators:


(4) recall=TP/(TP+FN)

(5) precision=TP/(TP+FP)

(6) specificity=T⁢N/(T⁢N+F⁢P)

(7) accuracy=(TP+TN)/(TP+FP+TN+FN)

(8) $$\mathrm{F1}=2*\frac{\mathrm{pression}*\mathrm{recall}}{\mathrm{pression}+\mathrm{recall}}$$

TP plus FN was the sample set of the actual spindles detected by experts, and TP plus FP was the sample set predicted as actual spindles by the detection algorithm. TP represents samples that were actually true spindle waves and detected by the automatic detection algorithm as true spindle waves; FP represents samples that were non-spindle waves but detected as true spindle waves; FN represents samples that were actually true spindle waves but predicted by the detection algorithm as non-spindle waves; and TN represents samples that are actually non-spindle waves and predicted by the detection algorithm as non-spindle waves.

## 3. Analysis of experimental results

The spindle detector automatically detected the spindle and contrasted it with the spindle detected by experts. R-spindles represented the actual spindle in the automatically detected spindles and A-spindles denoted all spindles automatically detected by the algorithm. The E-spindles symbolized the actual spindle detected by experts, and the intersections of the three experts’ two or three detection results were considered E-spindles.

Table 2 shows the number of spindle waves detected by the Morlet wavelet, windowed RMS, HMM&SVM, and hierarchical fusion algorithms compared to the real spindle waves labeled by experts. In Table 2, A-spindle represents the number of all spindle waves automatically detected by the algorithm, R-spindle represents the number of true spindle waves among the automatically detected spindle waves, and E-spindle represents the true spindle waves detected by the expert.

**TABLE 2:** Comparison of the results of different detection algorithms.

Subject	Morlet wavelet algorithm		RMS algorithm		HMM-SVM algorithm		Fusion algorithm		E-spindle
	**A-spindle**	**R-spindle**	**A-spindle**	**R-spindle**	**A-spindle**	**R-spindle**	**A-spindle**	**R-spindle**	
DS1	732	647	1409	998	1258	994	1124	1005	1136
DS2	467	424	831	602	802	685	725	668	730
DS3	263	229	518	383	617	527	511	472	551
DS4	231	204	589	406	523	436	456	429	493
DS5	439	376	977	747	821	765	792	763	848
DS6	184	160	632	491	635	532	530	487	590
DS7	702	605	1346	984	866	603	1063	994	1073
DS8	479	427	746	590	733	616	630	581	657
DS9	212	187	692	526	805	668	621	578	706
DS10	643	574	846	669	762	694	792	706	779
DS11	752	629	1415	1070	1238	1035	1248	1125	1265
DS12	525	481	968	708	774	688	834	761	819
DS13	596	528	724	571	729	605	634	583	676
DS14	381	334	898	639	880	763	759	702	798
DS15	1066	938	1635	1323	1458	1237	1507	1359	1491
DS16	539	456	736	608	664	503	687	643	736
DS17	551	492	939	712	875	756	835	765	839
DS18	572	489	929	674	802	701	790	688	763
DS19	360	324	727	598	768	654	689	640	702
DS20	736	635	965	763	1033	789	856	784	891
DS-average	521	457	926	703	852	712	804	737	827

As shown in Table 2, the total number of spindles automatically detected by the four methods differed for the same data. The four methods detected the most spindles in sample DS15, while the total spindles detected in sample DS6 were the least. The number of samples detected by the Morlet wavelet algorithm was small. Compared with the wavelet algorithm, the RMS-based algorithm could detect more spindles. The average number of R-spindles detected by HMM&SVM was 712, higher than the Morlet wavelet and RMS algorithm, demonstrating that this algorithm could recall more spindles.

Compared with the Morlet wavelet method, the R-spindle/A-spindle ratio of the hierarchical fusion algorithm was higher, reaching 91.67%, indicating that the hierarchical fusion algorithm improved the recall rate. Compared with the RMS algorithm and the HMM&SVM algorithm, the number of A-spindles of the hierarchical fusion algorithm decreased by 122 and 48, respectively, without a substantial change. The number of R-spindles did not decrease but instead increased by 34 and 25, respectively, showing that the accuracy of the newly proposed algorithm had been substantially enhanced.

The A-spindle of the hierarchical fusion algorithm had the recombined set of the two spindle detection results. After clustering, they had the Morlet, the RMS, and the non-recombined sets. The maximum number of spindles detected reached 1,507 in DS15 samples and 530 in DS6 samples, and the average value was 804. However, the maximum value of R-spindles reached 1,359 in DS15 samples, and the average value was 737. The fusion algorithm was closest to the actual spindle value detected by experts.

**FIGURE 4:**
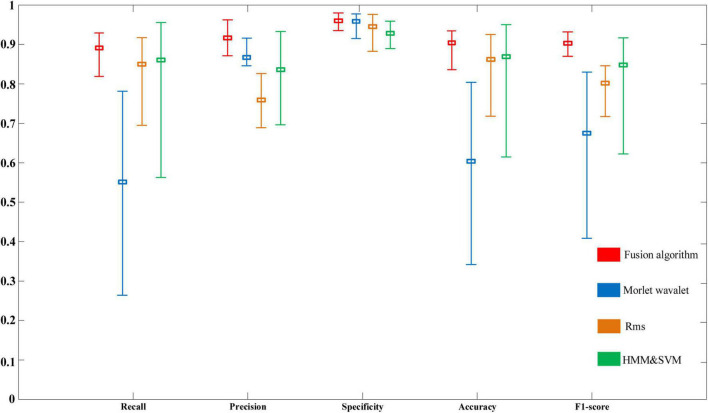
Performance comparison of detection algorithms based on subject data.

Figure 4 contrasts the hierarchical fusion algorithm with the other three automatic detection algorithms by calculating five performance evaluation indexes: Precision, Recall, Specificity, Accuracy, and F1-score.

The fusion algorithm represented the newly proposed hierarchical fusion algorithm, and Morlet wavelet, RMS, and HMM&SVM represented the Morlet wavelet, window RMS, and HMM and SVM, respectively. Figure 4 shows that the Precision of the spindle detection based on the Wavelet algorithm could meet the accuracy requirements under the current standard, scoring more than 85%. However, a critical gap existed between the maximum and minimum values of Recall, which had large fluctuations, and its stability needs to be improved. Only the evaluation index of Specificity of spindle automatic detection based on the RMS algorithm reached more than 90%. Likewise, the Specificity evaluation index of the HMM&SVM algorithm was more than 90% but lower than the other three detection algorithms. In the subject samples, the Recall index of the hierarchical fusion algorithm reached 92.9% at its highest, 81.9% at its lowest, and with an average of 89.1 ± 8.84%. In the performance of Precision, the maximum value reached was 96.3%, the minimum value was 87.1%, and the average value was 91.6 ± 3.06%. This Precision average value was better than those with the RMS and HMM&SVM algorithms and slightly better than those with the Morlet wavelet algorithm. The performances of Specificity, Accuracy, and F1-score were also ideal, and the average values were higher than those of the other three methods. The average values in the subject samples were 96 ± 2.89, 90.4 ± 6.50, and 90.3 ± 2.49%, respectively. The maximum, minimum, and average of each evaluation index of the hierarchical fusion algorithm were substantially improved compared with the other three algorithms.

## 4. Discussion

As sleep spindles are a characteristic index to evaluate sleep quality, the pursuit of the accurate detection of sleep spindles is imperative. The fusion method proposed in this paper was based on the Morlet wavelet and the Window RMS algorithm, combined with the improved k-means algorithm, and then the data results of algorithmic optimization. The fusion detection spindle algorithm combined the advantages of the three algorithms. Compared with previous research on spindle automatic detection, it improved calculation and effectiveness. The spindle detection results of the hierarchical fusion algorithm are shown in Figure 4, demonstrating better consistency with the expert detection results than the other methods. The detection rate of actual spindles was critically improved compared with the previous detection methods, effectively improving the accuracy and speed of the automatic spindle detection. This proposed method improved the shortcomings of the existing spindle detection methods and effectively enhances the detection efficiency of doctors, and reduces the visual inspection workload of sleep clinicians and the cost of detection (Jiang et al., 2021).

In the study of Warby et al. (2014), the amplitude and duration of the spindle decreased with age, probably damaging the spindle recognition performance. Therefore, in this experiment, we also paid attention to the interference of other unnecessary sample factors. PSG equipment was used to experiment on 20 subjects, and sleep data of 8 h were intercepted for research and analysis. The age of the subjects was controlled between 20 and 40 years old. The experimental data were truncated (Herrmann et al., 2016) to ensure a specific length of sample time. The wavelet automatic spindle detection method proposed by Wamsley et al. (2012) is more hierarchical than the fusion algorithm. The wavelet automatic detection algorithm needs much calculation and cumbersome experiments. The hierarchical fusion algorithm simplified the calculation, solving this problem. After completing the automatic spindle detection, the outliers and misjudged spindles were first eliminated. Then clustering processing was conducted to improve the effectiveness of spindle detection and simplify the process.

The number of actual spindles detected by the Wavelet algorithm proposed by Athanasios and Clifford (2015) was less than that detected by experts, so it cannot replace expert detection methods. This study used a sliding window to calculate the corrected moving average of the signal for the threshold setting. This method shortened the detection time and improved the accuracy. The spindles detected by the Martin et al. (2012) window root mean square method contained more false spindles. The hierarchical fusion algorithm clustered the non-coincident spindles automatically detected, improving the stability of spindle detection. The hierarchical fusion algorithm combined the Morlet wavelet and window RMS. It adopted the ideal accuracy of the wavelet method and the ideal recall rate of the window RMS detection method. Thus, the fusion algorithm realized both high levels of precision and recall and could achieve high evaluation indexes.

The HMM&SVM algorithm (Löfhede et al., 2008) also inspired the improvement of the algorithm in this paper. The influence of spindle amplitude on spindle detection was avoided, as the fluctuation range is too large to be ideal for the stability of the accuracy of the HMM&SVM algorithm. The hierarchical fusion algorithm advanced the experimental data to sample clustering, improving the detection speed through iterative clustering (Ding et al., 2018), and optimized the algorithm before clustering.

The figures reveal that the average Recall rate of the wavelet fusion method was 91.4%. The average Recall rate of the Precision method was 91.4%, which can be improved by 91.4% compared with that of the previous method, and the average Recall rate of the Precision method is improved by 90.4% compared with that of the Precision method. It met the requirements of improving the performance index and stability of the spindles.

Accurate and effective detection of sleep spindles is a methodological challenge. The spindles have a necessary judgment basis for diagnosing human diseases (Manoach et al., 2020). The hierarchical fusion algorithm is a favorable and feasible method for liberating the “gold standard” detection of experts, and reducing the shortcomings of the cumbersome, expensive, and strongly subjective spindle detection methods of the past (Parekh et al., 2017). This method could be popularized for clinical disease diagnosis instead of artificial spindle detection as it improves the speed of disease diagnosis and enables patients to receive rapid treatment (Imtiaz and Rodriguez-Villegas, 2014). At the same time, according to this test, the study of spindles on human intelligence and memory can save substantial experimental time (Wei et al., 2020). Therefore, effective and rapid spindle detection method is a common research direction.

The experiment mentioned in this paper only used the spindle samples of 20 subjects for analysis due to the limitation of the number and age of subjects and limited conditions; as a sample base, this is insufficient. In future research, increasing the sample base will improve the credibility of the results further. In the spindle detection algorithm, the spindle detection used in this paper was based on a single-channel C3, which uses too few channels. We could use dual-channel or multi-channels to detect the spindle automatically in future research. Concurrently, we could combine more deep learning models to classify the spindles and explore the prospects of deep learning in spindle classification.

## Data availability statement

The original contributions presented in this study are included in this article/supplementary material, further inquiries can be directed to the corresponding authors upon reasonable request, and according the policies of Tianjin University, Tianjin university of Technology, Tianjin Medical University General Hospital, Xuanwu Hospital, Capital Medical University.

## Ethics statement

The studies involving human participants were reviewed and approved by the Ethics Committee of Xuanwu Hospital. The patients/participants provided their written informed consent to participate in this study.

## Author contributions

CC and JM completed the ethics files of this experiment. CC and ZH recorded the original experiment data, analyzed the experiment data, and penned the manuscript. CC, LL, DM, and AB wrote parts of the manuscript. FL, WY, PL, and JL designed the experiment and revised the manuscript. All authors contributed to the article and approved the submitted version.
